# Kisspeptin Neurons in the Infundibular Nucleus of Ovariectomized Cats and Dogs Exhibit Unique Anatomical and Neurochemical Characteristics

**DOI:** 10.3389/fnins.2020.598707

**Published:** 2020-12-02

**Authors:** Éva Rumpler, Szabolcs Takács, Balázs Göcz, Ferenc Baska, Ottó Szenci, András Horváth, Philippe Ciofi, Erik Hrabovszky, Katalin Skrapits

**Affiliations:** ^1^Laboratory of Reproductive Neurobiology, Institute of Experimental Medicine, Budapest, Hungary; ^2^Department of Exotic Animal and Wildlife Medicine, University of Veterinary Medicine, Budapest, Hungary; ^3^Department of Obstetrics and Food Animal Medicine Clinic, University of Veterinary Medicine, Üllõ, Hungary; ^4^MTA-SZIE Large Animal Clinical Research Group, University of Veterinary Medicine, Üllõ, Hungary; ^5^INSERM U1215, Neurocentre Magendie, University of Bordeaux, Bordeaux, France

**Keywords:** arcuate nucleus, carnivores, hypothalamus, KNDy neurons, neurokinin B, neuropeptides, reproduction

## Abstract

Neurons co-synthesizing kisspeptin (KP), neurokinin B (NKB), and dynorphin (“KNDy neurons”) in the hypothalamic arcuate/infundibular nucleus (INF) form a crucial component of the gonadotropin-releasing hormone (GnRH)/luteinizing hormone (LH) “pulse generator.” The goal of our study was to characterize KP neuron distribution, neuropeptide phenotype and connectivity to GnRH cells in ovariectomized (OVX) dogs and cats with immunohistochemistry on formalin-fixed hypothalamic tissue sections. In both species, KP and NKB neurons occurred in the INF and the two cell populations overlapped substantially. Dynorphin was detected in large subsets of canine KP (56%) and NKB (37%) cells and feline KP (64%) and NKB (57%) cells; triple-labeled (“KNDy”) somata formed ∼25% of all immunolabeled neurons. Substance P (SP) was present in 20% of KP and 29% of NKB neurons in OVX cats but not dogs, although 26% of KP and 24% of NKB neurons in a gonadally intact male dog also contained SP signal. Only in cats, cocaine- and amphetamine regulated transcript was also colocalized with KP (23%) and NKB (7%). In contrast with reports from mice, KP neurons did not express galanin in either carnivore. KP neurons innervated virtually all GnRH neurons in both species. Results of this anatomical study on OVX animals reveal species-specific features of canine and feline mediobasal hypothalamic KP neurons. Anatomical and neurochemical similarities to and differences from the homologous KP cells of more extensively studied rodent, domestic and primate species will enhance our understanding of obligate and facultative players in the molecular mechanisms underlying pulsatile GnRH/LH secretion.

## Introduction

Pulsatile secretion of hypothalamic gonadotropin-releasing hormone (GnRH) and adenohypophysial luteinizing hormone (LH) is crucial for mammalian fertility (Belchetz et al., 1978; Santoro et al., 1986). Evidence accumulated over the past two decades indicates that two populations of kisspeptin (KP) synthesizing neurons located in the preoptic region and in the arcuate (ARC)/infundibular (INF) nucleus of the mediobasal hypothalamus, respectively, are key regulators of GnRH/LH secretion. Kisspeptin stimulates LH release (Gottsch et al., 2004; Dhillo et al., 2005; Messager et al., 2005; Plant et al., 2006; Albers-Wolthers et al., 2014, 2016) via directly activating GnRH neurons, which express the KP receptor (Kiss1r) (Irwig et al., 2004; Han et al., 2005; Messager et al., 2005). This mechanism is so basic for reproduction that ablation of *Kiss1r* from GnRH neurons causes infertility, and conversely, fertility can be reinstated to mice bearing global *Kiss1r* deletion via the selective reinsertion of the *Kiss1r* into GnRH neurons (Kirilov et al., 2013).

While preoptic KP neurons are implicated in positive sex steroid feedback and the mid-cycle GnRH/LH surge (Herbison, 2008), KP neurons of the hypothalamic ARC/INF are important regulators of negative sex steroid feedback (Mittelman-Smith et al., 2012) and form key component of the long-sought GnRH/LH pulse generator network (Clarkson et al., 2017). Arcuate KP neurons, widely referred to as “KNDy” neurons (Lehman et al., 2010a), co-synthesize neurokinin B (NKB) and dynorphin (Dyn) in ovine, caprine, and rodent species (Burke et al., 2006; Ciofi et al., 2006; Foradori et al., 2006; Goodman et al., 2007; Navarro et al., 2009; Cheng et al., 2010; Wakabayashi et al., 2010). Abundant network communication of these cells with each other (Burke et al., 2006; Foradori et al., 2006; Goodman et al., 2007; Navarro et al., 2009; Cheng et al., 2010; Wakabayashi et al., 2010) uses multiple peptidergic mechanisms which are critical for shaping the GnRH/LH secretory pulses (Navarro et al., 2009). In mice, excitatory NKB neurotransmission via NK3 autoreceptors and inhibitory Dyn signaling via κ-opioid autoreceptors play crucial roles in this communication (Navarro et al., 2011; de Croft et al., 2013; Ruka et al., 2013; Qiu et al., 2016).

While KP itself does not change the electric activity of KNDy neurons (de Croft et al., 2013), it provides the major output signal from the KNDy neuron network to GnRH neurons which express the *Kiss1r* (Irwig et al., 2004; Han et al., 2005; Messager et al., 2005).

Against the common assumption that the molecular mechanism of pulsatile GnRH/LH secretion are conserved across mammals, some unexpected species differences have been noticed with respect to the neuropeptide composition of ARC/INF KNDy neurons. Likewise, while NKB has been observed in KP neurons of all species investigated so far, this colocalization is far less complete in humans than suggested earlier for sheep (Goodman et al., 2007) or mice (Navarro et al., 2009) and depends significantly on the sex and age of subjects (Hrabovszky et al., 2011, 2012; Molnar et al., 2012). Accordingly, only ∼36% of NKB neurons express KP immunoreactivity in young human males, which increases to ∼69% in males aged above 50 years (Molnar et al., 2012). In addition, Dyn, which may be present in virtually all KP neurons in sheep (Goodman et al., 2007) and mice (Navarro et al., 2009), can only be visualized very rarely with immunohistochemistry in human KP cells (Hrabovszky et al., 2012, 2019; Skrapits et al., 2015a). Similarly, galanin exhibits a high-degree of colocalization with KP in mice, but not in humans (Skrapits et al., 2015a). To the contrary, postmenopausal women show substance P (SP) immunoreactivity in ∼25% and ∼31% of KP and NKB neurons, respectively (Hrabovszky et al., 2013), whereas these instances of colocalization have not been reported in rodents and stated as non-existing in non-human primates (Kalil et al., 2016). Further, cocaine- and amphetamine-regulated transcript (CART) is present in ∼48% of KP-IR and ∼30% of NKB-IR perikarya in postmenopausal women and also colocalized with KP in non-human primates (True et al., 2017), but not in rodents (True et al., 2013). The above species differences suggest that some co-expressed neuropeptides can play obligate roles in the molecular mechanisms of episodic GnRH/LH secretion, whereas the contribution of others might be facultative.

Additional species-specific features in the neuroanatomy of the pulse generator include the variable topography of GnRH neurons (King and Anthony, 1984) and the relative importance of the ARC/INF KP cell population as a source for KP fibers innervating the somatodendritic compartment of GnRH neurons. Likewise, GnRH neurons in mice are located in the preoptic area and only ∼4% of their KP inputs contain NKB, a distinctive marker of KP fibers originating in the ARC (Kallo et al., 2012). In contrast, GnRH neurons in humans are widely distributed throughout the hypothalamus (Dudas et al., 2000) and at least 10–30% of their KP input originates in the INF and thus, contain both KP and NKB (Hrabovszky et al., 2011; Molnar et al., 2012).

Anatomical comparison of phylogenetically distant mammalian species can provide deeper insight into common and variable operative mechanisms of the GnRH/LH pulse generator. Here we chose to investigate mediobasal hypothalamic KP neuron topography, neurochemistry and connectivity to GnRH neurons in two poorly studied carnivore species, the dog and the cat.

## Materials and Methods

### Statement of Ethics

All studies were carried out with the permission from the Animal Welfare Committee of the Institute of Experimental Medicine (No. MAB 1/2016) and in accordance with legal requirements of the European Community (Decree 86/609/EEC).

### Section Preparation for Immunohistochemistry

Tissue samples were collected from the brain of four ovariectomized (OVX) dogs, one gonadally intact male dog and six OVX cats (for details, see Table 1). All pets were euthanized by a veterinarian because of incurable and severe health issues. The cadavers were decapitated and, following the removal of the calvaria, the brain was removed and immersed immediately into 4% formaldehyde in 0.1 M phosphate buffered saline (PBS; pH 7.4) for 10 days. Hypothalamic blocks were dissected out, infiltrated with 20% sucrose, and sectioned at 30 μm with a Leica SM 2000R freezing microtome (Leica Microsystems, Nussloch Gmbh, Germany). The sections were stored permanently in cryoprotective solution (30% ethylene glycol; 25% glycerol; 0.05 M phosphate buffer; pH 7.4) at −20°C before immunohistochemical processing.

**TABLE 1:** Basic data on canine and feline tissue samples and their use in different experiments.

	Animals		Sex	Age (yrs)	Gonadal state	Used in mapping studies		Used in colocalization studies
						
						KP	GnRH	
Dogs	D#1	mixed-breed	♀	1.5	OVX			+
	D#2	mixed-breed	♀	N/A	OVX	+	+	+
	D#3	mixed-breed	♀	12	OVX	+	+	+
	D#4	vizsla	♀	17	OVX	+	+	+
	D#5	beagle	♂	11.5	intact	+		+
Cats	C#1		♀	N/A	OVX			+
	C#2		♀	9.5	OVX	+		+
	C#3		♀	N/A	OVX	+	+	+
	C#4		♀	12	OVX	+	+	+
	C#5		♀	aged	OVX			+
	C#6		♀	N/A	OVX	+	+	+

### Experiment 1. Mapping the Distribution of Canine and Feline Kisspeptin Neurons and GnRH Cells

To study the topography of canine and feline KP and GnRH neurons, every 12–24th hypothalamic section was used from each brain. The sections were rinsed in PBS for 3 × 5 min and pretreated with a mixture of 3% H_2_O_2_ and 0.5% Triton X-100 for 30 min, followed by antigen retrieval with 0.1 M citrate buffer (pH 6.0) at 80°C for 30 min. The sections were incubated overnight at room temperature in different polyclonal antibodies against GnRH or KP (Table 2), followed by biotinylated secondary antibodies (Jackson ImmunoResearch Laboratories, West Grove, PA, United States; 1:500, 60 min) and the ABC Elite reagent (Vector, Burlingame, CA, United States; 1:1,000, 60 min). The peroxidase signal was developed with nickel-intensified diaminobenzidine chromogen. The sections were mounted on microscope slides from Elvanol, air-dried, dehydrated with 70 and 95% (5 min each), followed by 100% (2 × 5 min) ethanol, cleared with xylene (2 × 5 min) and coverslipped with DPX mounting medium (Merck Group, Darmstadt, Germany). Representative light microscopic images were prepared with an AxioCam MRc 5 digital camera mounted on a Zeiss AxioImager M1 microscope, using the AxioVision 4.6 software (Carl Zeiss, Göttingen, Germany). The distribution of GnRH neurons was illustrated with green triangles, each corresponding to a single GnRH neuron of a representative animal. Ten KP neurons found in a given region of a representative animal were illustrated with a red dot. Schemas were created with PowerPoint using modified canine and feline coronal atlas plates from BrainMaps.org (Mikula et al., 2007).

**TABLE 2:** Specification, source and dilution of primary antibodies used in immunohistochemical and immunofluorescence experiments.

Antigen	Host species	Dilution		Source/characterization
		
		Studies in dogs	Studies in cats	
GnRH	Guinea pig	1:50,000 (IHC); 1:5,000 (IF)	1:50,000 (IHC); 1:5,000 (IF)	EH#1018, Hrabovszky et al., 2011
GnRH	Rat	1:2,000 (IF)	1:2,000 (IF)	EH#1044, Skrapits et al., 2015b
KP-10	Rabbit	1:75,000 (IHC, IF-TSA)	1:200,000 (IHC)	AC566, Franceschini et al., 2006
KP-10	Sheep	x	1:5,000 (IF)	AC024, Caraty et al., 1980; Porteous et al., 2011
rat proNKB	Guinea pig	1:30,000 (IF-TSA)	x	IS-3/61; Ciofi et al., 1994
rat proNKB	Guinea pig	x	1:30,000 (IF-TSA)	IS-3/63; gift from P. Ciofi
DynB	Rabbit	1:1,000 (IF)	1:30,000 (IF-TSA)	IS-35; Ciofi et al., 2006
SP	Rat	1:1,000 (IF)	1:1,000 (IF)	8450-0505; Abd Serotec
CART	Mouse	1:30,000 (IF-TSA)	1:30,000 (IF-TSA)	Vrang et al., 1999
galanin	Sheep	1:100,000 (IF-TSA)	1:100,000 (IF-TSA)	MS-FJL 17-3, Lopez et al., 1990

*CART, cocaine- and amphetamine-regulated transcript; DynB, dynorphin B; GnRH, gonadotropin-releasing hormone; IF, immunofluorescence; IHC, immunohistochemistry; KP, kisspeptin; NKB, neurokinin B; SP, substance P; TSA, tyramide signal amplification.*

### Experiment 2. Colocalization Studies of Neuropeptide Cotransmitters in Canine and Feline Kisspeptin Neurons

To address the expression of Dyn, SP, CART, and galanin in canine and feline KP and NKB neurons, four separate triple-immunofluorescent experiments were carried out. After section pretreatments, the first neuropeptide antigen was visualized using sequential incubation in polyclonal primary antibodies (48 h, 4°C), followed by biotinylated secondary antibodies (Jackson ImmunoResearch; 1:500, 120 min), ABC Elite reagent (Vector; 1:1,000, 60 min) and finally, Cy3-tyramide (1:1,000 in 0.05 M Tris-HCl buffer, pH 7.6, containing 0.005% H_2_O_2_, 30 min) (Hrabovszky et al., 2013). Then, horseradish peroxidase was inactivated with 1% H_2_O_2_ for 30 min and the sections were incubated in a cocktail of two additional primary antibodies (48 h, 4°C) to detect the remaining two neuropeptides. One of these was visualized using peroxidase-conjugated secondary antibodies (Jackson ImmunoResearch; 1:250, 120 min), followed by FITC-tyramide (1:1,000 in 0.05 M Tris-HCl buffer, pH 7.6, containing 0.005% H_2_O_2_) for 30 min. The third primary antibody was detected with Cy5-conjugated secondary antibodies (Jackson ImmunoResearch; 1:500, 120 min). Specification, source and optimal dilution of primary antibodies are provided in Table 2. One triple-labeling experiment had to use two primary antibodies raised in the same host species. As in a former KP study (Goodman et al., 2007), the strategy to eliminate cross-reaction of secondary antibodies was adapted from a previous technical report (Hunyady et al., 1996). First, the rabbit anti-KP-10 antibodies (#566, Caraty, Franceschini et al., 2006) were applied to the sections at an extremely low concentration (1:75,000) and visualized using biotinylated secondary antibodies (Jackson ImmunoResearch; 1:500, 120 min), ABC Elite reagent (Vector; 1:1,000, 60 min) and finally, Cy3-tyramide (1:1,000 in 0.05 M Tris-HCl buffer, pH 7.6, containing 0.005% H_2_O_2_, 30 min). Subsequently, the rabbit anti-DynB (IS-35; Ciofi et al., 2006) antibodies were used at a high concentration (1:1,000), and visualized with Cy5-conjugated anti-rabbit secondary antibodies (Jackson ImmunoResearch; 1:500, 120 min). To verify that the Cy5-conjugated antibodies do not bind to the anti-KP-10 antibodies, control experiments were carried out in parallel. Cy5-conjugated anti-rabbit antibodies did not produce any Cy5 signal in control sections preincubated with highly diluted anti-KP-10 antibodies (#566, 1:75,000), indicating that the KP/DynB colocalization strategy with two rabbit primary antibodies was devoid of non-specific Dyn signal.

In each triple-labeling assay, Z-projected confocal slices were analyzed to study neuropeptide co-expression in KP-IR and NKB-IR perikarya. Representative photomicrographs (1-4/animal) were prepared from triple-labeled sections of the INF of 2-5 dogs and 4 cats. ∼200–900 labeled neurons were analyzed in each experiment and the extents of colocalization were determined separately in each animal and expressed as Mean ± SEM of the animals.

### Experiment 3. Analysis of the Connectivity Between Canine and Feline Kisspeptin Neurons and GnRH Cells

Triple-immunofluorescent labeling for KP, NKB and GnRH was carried out to visualize KP and NKB inputs to the somatodendritic compartment of GnRH neurons. The number of KP and NKB contacts were counted on 7–14 randomly selected GnRH neurons from each of 3 OVX dogs and 3 OVX cats and results were expressed as the Mean ± SEM. Appositions were defined as contacts with no gap between the juxtaposed profiles in confocal slices and illustrated in orthogonal side-views of z-stacks. Considering the absence of sharp transition between the somatic and dendritic compartments in typical GnRH neurons, contacts identified on GnRH-IR profiles thinner than 5 μm were arbitrarily categorized as axodendritic. The total length of GnRH dendrites analyzed from dogs and cats were 1160.3 and 1787.7 μm, respectively. The frequency of dendritic afferents was expressed by dividing the number of contacts with the dendrite length of each animal and expressed as Mean ± SEM. Presence of axo-axonal appositions between KP and GnRH fibers was assessed qualitatively via studying the median eminence (ME).

### Confocal Microscopy

The immunofluorescent specimens were mounted on glass slides from 0.1M Tris-HCl buffer (pH 7.6) and cover slipped with the aqueous mounting medium Mowiol. Signals were studied with a Zeiss LSM780 confocal microscope. High-resolution images were captured using a 20×/0.8 NA objective, a 0.6–1× optical zoom and the Zen software (CarlZeiss). In innervation studies, a 63×/1.4 NA objective lens with a 1–2.3× optical zoom was used. Different fluorochromes were detected with the following laser lines: 488 nm for FITC, 561 nm for Cy3, 633 nm for Cy5. Emission filters were as follows: 493–556 nm for FITC, 570–624 nm for Cy3 and 638–759 nm for Cy5. To prevent emission crosstalk between the fluorophores, the red channel (Cy3) was recorded separately from the green (FITC)/far-red (Cy5) channels (“smart setup” function). To illustrate the results, confocal Z-stacks (Z-steps: 0.85–1 μm, pixel dwell time: 0.79–1.58 μs, resolution: 1024 × 1024 pixels, pinhole size: set at 1 Airy unit) were merged using maximum intensity Z-projection (ImageJ). Orthogonal side-views were created by Zen software. The final figures were adjusted in Adobe Photoshop and saved as TIF files.

## Results

### Experiment 1. Topography of Canine and Feline Kisspeptin Neurons and GnRH Cells

#### Distribution of Kisspeptin-Immunoreactive Neurons in the Infundibular Nucleus

**FIGURE 1:**
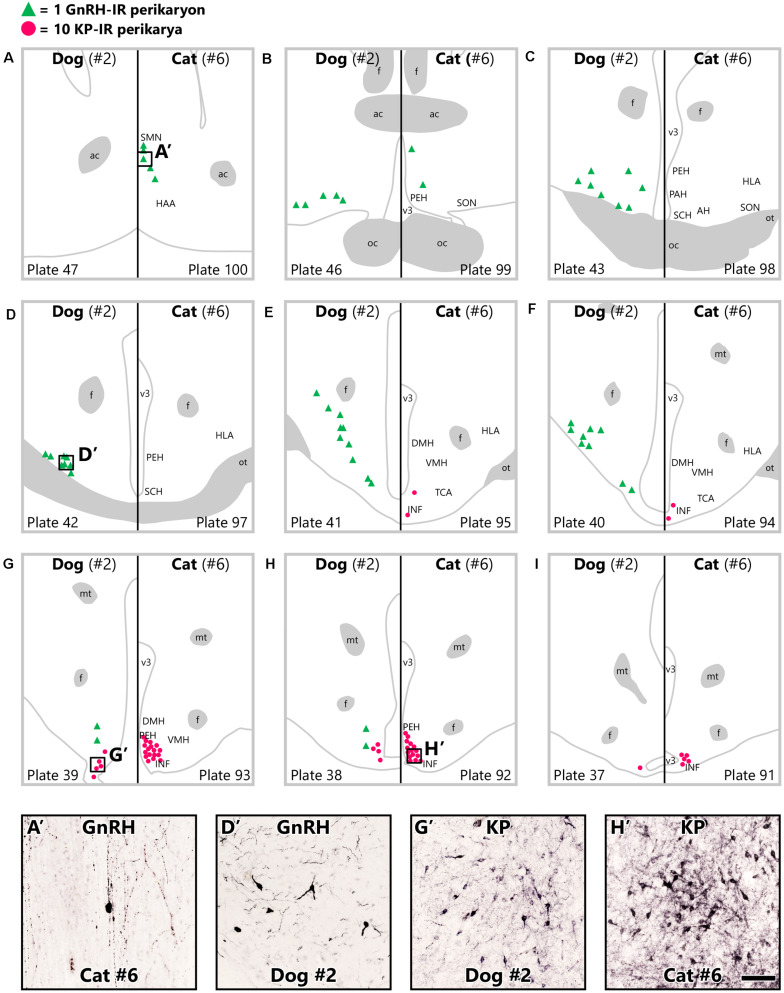
Topography of gonadotropin-releasing hormone and kisspeptin neurons in ovariectomized dogs and cats. Gonadotropin-releasing hormone (GnRH) neurons (green triangles) are scattered throughout the hypothalamus in ovariectomized (OVX) dogs and restricted to the septal-preoptic region in OVX cats. While kisspeptin-immunoreactive (KP-IR) neurons (red dots) are concentrated in the caudal infundibular nucleus in OVX cats as well as dogs, they also occur somewhat more rostrally in the feline mediobasal hypothalamus. Serial coronal schemas were **(A–I)** obtained from BrainMaps: An Interactive Multiresolution Brain Atlas (BrainMaps.org) (Mikula et al., 2007). Each green triangle corresponds to a single GnRH neuron and each red dot represents 10 KP-IR somata from animals D#2 and C#6. Representative photomicrographs at the bottom display immunolabeled GnRH and KP neurons from OVX cats **(A’,H’)** and dogs **(D’,G’)**, respectively. ac, anterior commissure; AH, anterior hypothalamic nucleus; DMH, dorsomedial hypothalamic nucleus; f, fornix; HAA, anterior hypothalamic area; HLA, lateral hypothalamic area; INF, infundibular nucleus; mt, mammillothalamic tract; oc, optic chiasm; ot, optic tract; PAH, paraventricular nucleus of the hypothalamus; PEH, periventricular complex of the hypothalamus; SCH, suprachiasmatic nucleus; SMN, medial septal nucleus; SON, supraoptic nucleus; TCA, area of the tuber cinereum; v3, third cerebral ventricle; VMH, ventromedial hypothalamic nucleus. Scale bar: 100 μm.

Immunohistochemical studies of tissue sections from different rostro-caudal levels of the hypothalamus (Figures 1A–I) visualized numerous heavily labeled KP-immunoreactive (IR) perikarya in the canine and feline mediobasal hypothalami (Figures 1E–I). The majority of these neurons were concentrated in the caudal part of the INF in both dogs (Figure 1G’) and cats (Figure 1H’). In OVX cats, fewer perikarya also occurred in the rostral INF and occasionally, in the periventricular nucleus. In both species, KP-IR fibers formed dense plexuses in the septal-preoptic region, the periventricular nucleus, the INF, the ventromedial and dorsomedial hypothalamic nuclei and the ME.

#### Differential Distribution of Canine and Feline GnRH Neurons

While KP cells displayed similar basic topography in the canine and feline mediobasal hypothalami with relatively minor species differences, the distribution of GnRH neurons considerably differed between the two species (Figures 1A–H). Accordingly, the distribution of canine GnRH neurons covered wide areas from the preoptic region to the mediobasal hypothalamus (Figures 1B–H), including the supraoptic nucleus, the medial preoptic area, the ventrolateral preoptic nucleus, the lateroanterior hypothalamic nucleus (Figure 1D’), the area of the tuber cinereum-lateral hypothalamic area continuum, the medial tuberal nucleus and the INF. In contrast, GnRH cells of the cat exhibited a much more restricted distribution in the septal-preoptic area (Figures 1A,B), including the medial septal nucleus (Figure 1A’) and the periventricular complex of the hypothalamus; only few scattered neurons were observed in the INF. The bulk of GnRH-IR fibers targeted the ME in both species.

### Experiment 2. Neuropeptide Phenotype of Canine and Feline Kisspeptin Neurons

#### Partial Overlap of Neurokinin B and Dynorphin Neurons With Kisspeptin Neurons

**FIGURE 2:**
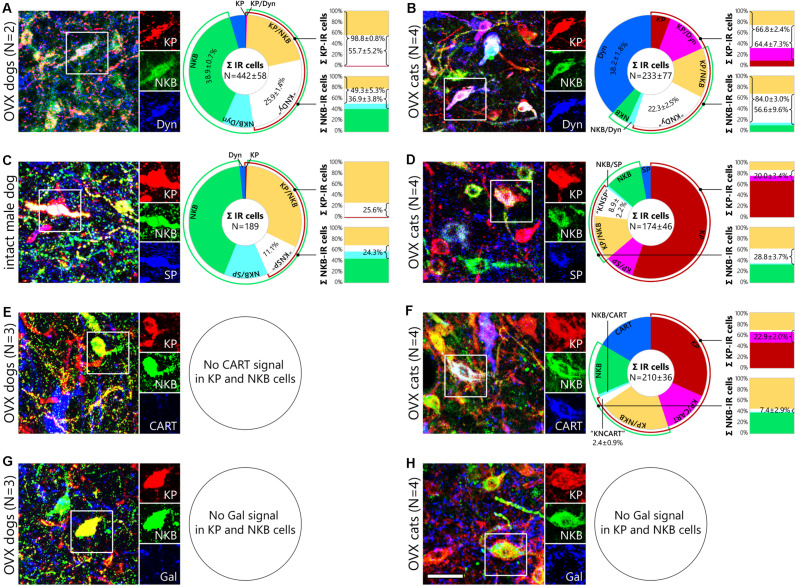
Neurochemistry of canine and feline kisspeptin and neurokinin B cells. Representative photomicrographs illustrate the neuropeptide phenotype of canine and feline kisspeptin (KP; red) and neurokinin B (NKB; green) neurons. Cells framed in the three-color figures are shown in separate color channels on the right side of each panel. KP and NKB immunoreactivities overlap considerably but incompletely in the infundibular nucleus of dogs **(A,C,E,G)** and ovariectomized (OVX) cats **(B,D,F,H)**. Pie charts illustrate the percent distribution of distinct neuronal phenotypes from triple-immunofluorescence experiments addressing dynorphin (Dyn; **A**), substance P (SP; **B**) and cocaine- and amphetamine-regulated transcript (CART; **C**) co-expression in KP and NKB neurons. Bar graphs show percentages of neuropeptide colocalization in KP neurons (upper columns) and NKB neurons (bottom columns). Dynorphin signal (blue) occurs in large subsets of canine **(A)** and feline **(B)** KP and NKB cells; ∼25% of the immunolabeled infundibular neurons are triple-labeled (“KNDy”) cells in both dogs and cats. Substance P (blue) is detectable in 26% of KP and 24% of NKB neurons in an intact male dog **(C)** as well as in 20% of KP and 29% of NKB cells in OVX cats **(D)**. CART (blue) is also present in 23% of KP and 7% of NKB neurons in OVX cats **(F)**, but no CART signal is detectable in canine KP and NKB cells **(E)**. Galanin is not expressed in KP and NKB neurons of either species (**G,H**). CART, cocaine- and amphetamine regulated transcript; Dyn, dynorphin; Gal, galanin; KP, kisspeptin; “KNCART”, kisspeptin/neurokinin B/cocaine- and amphetamine regulated transcript-positive cells; “KNDy”, kisspeptin/neurokinin B/dynorphin-positive cells; “KNSP”, kisspeptin/neurokinin B/substance P-positive cells; NKB, neurokinin B; SP, substance P. Scale bar: 50 μm.

Triple-immunofluorescent studies provided evidence for the presence of both NKB and Dyn in canine and feline KP neurons (Figures 2A,B). The quantitative analysis of 833 immunoreactive cell bodies from two OVX dogs (D#1, D#2) and 931 immunoreactive somata from four OVX cats (C#3, C#4, C#5, C#6) identified Dyn signal in ∼60% of KP neurons in both species (55.7 ± 5.2% in dogs, Figure 2A, upper column; 64.4 ± 7.3% in cats, Figure 2B, upper column), in 36.9 ± 3.8% of NKB neurons in dogs (Figure 2A, bottom column) and in 56.6 ± 9.6% of NKB neurons in cats (Figure 2B, bottom column). 66.8 ± 2.4% of KP perikarya contained NKB (Figure 2B, upper column) and 84.0 ± 3.0% of NKB neurons contained KP in cats (Figure 2B, bottom column), whereas virtually all (98.8 ± 0.8%) KP neurons expressed NKB (Figure 2A, upper column) and 49.3 ± 5.3% of NKB neurons expressed KP (Figure 2A, bottom column) in dogs. Triple-labeled (“KNDy”) somata contributed to 25.9 ± 1.4% and 22.3 ± 2.5% of the labeled neurons in dogs and cats, respectively (white slices of pie charts in Figures 2A,B). Interestingly, the most frequently encountered phenotypes were NKB-only neurons in dogs (38.9 ± 0.7%; Figure 2A, green slice of pie chart), and Dyn-only neurons in cats (38.2 ± 1.8%; Figure 2B, blue slice of pie chart).

#### Substance P Synthesis in Canine and Feline Kisspeptin and Neurokinin B Neurons

The putative expression of SP in KP and NKB neurons was addressed in additional triple-immunofluorescent experiments. Only two (D#1, D#2) out of the four OVX dogs contained SP signal in KP and NKB cells and SP was present in less than 2% of these cells. In contrast, quantitative analysis of 189 immunolabeled perikarya from a gonadally intact male dog (D#5) revealed SP signal in 25.6% of KP neurons (Figure 2C, upper column) and 24.3% of NKB neurons (Figure 2C, bottom column); 11.1% of the labeled neurons were triple-labeled (white slice of the pie chart in Figure 2C). As opposed to OVX dogs, OVX cats (C#2, C#3, C#4, C#6) contained SP signal in 20.0 ± 3.4% of KP perikarya (Figure 2D, upper column) and 28.8 ± 3.7 of NKB perikarya (Figure 2D, bottom column); 8.9 ± 2.2% of the 695 immunolabeled neurons were KP/NKB/SP-positive (white slice of pie chart in Figure 2D).

#### Cocaine- and Amphetamine Regulated Transcript in Kisspeptin and Neurokinin B Neurons of Cats but Not Dogs

Analysis of sections triple-labeled for KP, NKB and CART provided evidence that subpopulation of feline KP and NKB neurons also synthesize CART. The quantitative analysis of 838 cell bodies from four OVX cats (C#1, C#2, C#4, C#6) provided evidence for the presence of CART in 22.9 ± 2.0% of KP-IR (Figure 2F, upper column) and 7.4 ± 2.9% of NKB-IR (Figure 2F, bottom column) neurons; triple-labeled somata contributed to 2.4 ± 0.9% of the IR perikarya (white slice of pie chart in Figure 2F). CART was not detectable in canine KP or NKB neurons (Figure 2E).

#### Absence of Galanin in Kisspeptin and Neurokinin B Cells

Galanin, which was previously detected in KP neurons of mice (Porteous et al., 2011; Kallo et al., 2012) but not humans (Skrapits et al., 2015a), was not present in KP and NKB cells of OVX dogs (D#2, D#3, D#4) or cats (C#3, C#4, C#5, C#6) (Figures 2G,H).

### Experiment 3. Kisspeptin Innervation of GnRH Cells

#### Kisspeptin Inputs to the Somatodendritic Compartment of GnRH Neurons

**FIGURE 3:**
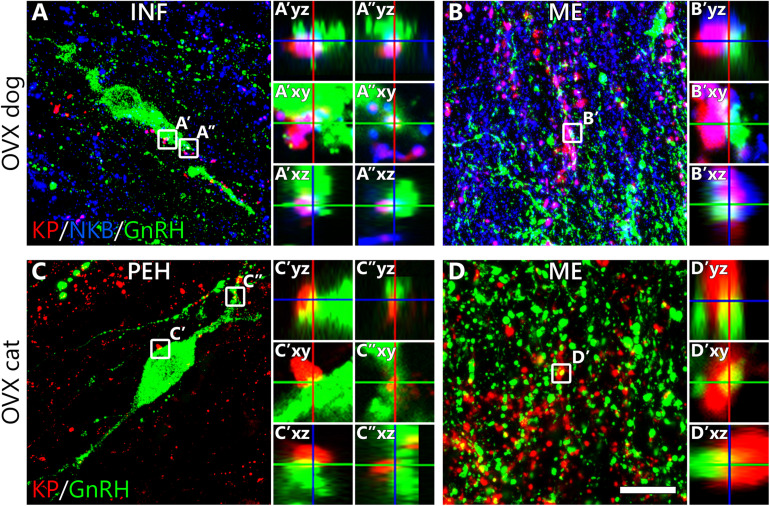
Kisspeptin innervation of canine and feline gonadotropin-releasing hormone neurons. Results of triple- and dual-immunofluorescent studies on ovariectomized dogs **(A,B)** and cats **(C,D)**, respectively, reveal that kisspeptin (KP) fibers (red) establish axo-somatic **(A’,C’)**, axo-dendritic **(A”,C”)** and axo-axonal **(B’,D’)** contacts with gonadotropin-releasing hormone (GnRH)-immunoreactive (green) neurons. Successful detection of neurokinin B (NKB) in fibers of the canine hypothalamus allows the identification of KP fibers that arise from KP/NKB dual-phenotype neurons of the infundibular nucleus. High-power images of framed regions show absence of gaps between the juxtaposed profiles in orthogonal side-views of contacts. INF, infundibular nucleus; ME, median eminence; PEH, periventricular complex of the hypothalamus; Scale bar: 20 μm in panels **(A,C)** (4 μm in insets), 16 μm in panels **(B,D)** (3 μm in insets).

In OVX cats (C#3, C#4, C#6), KP neurons sent dense projections to the septal-preoptic region where most GnRH neurons were located. Virtually all (94.4 ± 5.6%) GnRH neurons received at least one KP-IR input; appositions were observed on both the somatic (6.9 ± 0.2 inputs/soma, Figure 3C’) and the dendritic (7.5 ± 1.4 contacts/100 μm dendrite, Figure 3C”) compartments. The feline hypothalamus showed very poor axonal labeling for the INF KP neuron markers NKB or Dyn, making it impossible to assess whether and to what extent, KP afferents to GnRH neurons arise from the INF. In OVX dogs (D#4, D#5, D#6), 77.6 ± 17.6% of GnRH neurons (75.8 ± 18.3% in the preoptic region and 83.3 ± 16.7% in the INF) were innervated by at least one KP fiber and GnRH neurons in the INF also received more KP-IR contacts than those located in more rostral hypothalamic regions [6.3 ± 1.0 (Figure 3A’) vs. 2.0 ± 0.7 inputs/soma and 7.9 ± 5.5 (Figure 3A”) vs. 0.9 ± 0.6 inputs/100 μm dendrite]. 57.6 ± 14.1% of inputs (93.0 ± 1.5% in the INF) targeting the somatodendritic compartment were also immunopositive for NKB, indicating that more than half of these KP afferents arise from the INF (Figure 3A).

#### Axo-axonal Communication Between Kisspeptin and GnRH Neurons in the Median Eminence

The distribution of hypophysiotropic GnRH fibers in the ME overlapped with a dense KP-IR fiber plexus in both dogs and cats (Figures 3B,D). Contacts suggesting axo-axonal interactions were often detected between the juxtaposed GnRH and KP profiles. In dogs, many KP fibers contained NKB signal as well, indicating their mediobasal hypothalamic origin (Figure 3B).

## Discussion

The topography of GnRH and KP neurons and the peptide neurochemistry of the putative pulse generator KP neurons within the mediobasal hypothalamus differ substantially between the most widely studied laboratory rodent models and humans. Although the GnRH/LH pulse generator function is thought to be conserved across mammals, differences indicate that the anatomical basis and molecular mechanisms of episodic GnRH secretion can vary between species. Here we carried out the neuroanatomical characterization of KP and GnRH neurons in OVX dogs and cats. Information about the pulse generator network of these two poorly studied carnivore species may provide important insight into evolutionarily conserved *vs.* variable mechanisms of episodic GnRH/LH secretion.

### Distribution of GnRH and Kisspeptin Neurons in Dogs and Cats

GnRH neurons are not concentrated within distinct hypothalamic nuclei and their distribution varies considerably between species (King and Anthony, 1984). For example, while GnRH cell bodies are located exclusively within the septal-preoptic region in laboratory rodents (Merchenthaler et al., 1980), in humans they are scattered quite widely from the septo-preoptic region to the retromammillary area (Dudas et al., 2000; Hrabovszky and Liposits, 2013). Similarly to human, OVX dogs used in our study contained GnRH-IR perikarya throughout the hypothalamus, including the INF. The functional difference between the rostral and the caudal GnRH cell groups of this species requires clarification. In sheep, both the preoptic and the mediobasal hypothalamic GnRH neuron populations express Fos during the LH surge (Moenter et al., 1993), whereas Fos only occurs in GnRH neurons of the ARC at times of enhanced pulsatile GnRH/LH secretion (Boukhliq et al., 1999).

In contrast with dogs, OVX cats contained the majority of GnRH neurons in the septal-preoptic region. This observation is in line with the previously reported distribution of GnRH-immunopositive perikarya in cats (Barry and Dubois, 1975; Mikami et al., 1985; Belda et al., 2000), except that in one study adult male (Belda et al., 2000) cats also contained GnRH cell bodies in the INF.

A variety of mammals, including humans (Hrabovszky et al., 2010; Rumpler et al., 2020), contain two major KP cell populations in the preoptic region and in the ARC/INF of the mediobasal hypothalamus, respectively (Lehman et al., 2010b). An additional small group of KP neurons has been identified recently in the medial amygdala of rodents (Stephens and Kauffman, 2017). In rats and mice, preoptic KP neurons play important roles in positive estrogen feedback and the mid-cycle GnRH/LH surge (Herbison, 2008). *Kiss1* expression in these cells is regulated positively by estrogens (Adachi et al., 2007). The largest KP cell group in all species studied so far has been localized to the ARC/INF of the mediobasal hypothalamus (Lehman et al., 2010b); these neurons have been implicated in negative sex steroid feedback (Mittelman-Smith et al., 2012) and their *Kiss1* expression is inhibited by gonadal sex steroid hormones (Smith et al., 2005). There is accumulating evidence that KP neurons of the ARC/INF play a critical role in the generation of GnRH/LH pulses (Clarkson et al., 2017).

Our mapping experiments localized the majority of KP neurons to the INF of OVX dogs and cats. In addition, the feline, but not the canine hypothalamus contained scattered IR neurons in the periventricular nucleus, in line with results of a recent immunohistochemical study by Amelkina et al. (2019). These authors studied intact female cats and identified two additional KP cell populations in the anterior periventricular nucleus and the amygdaloid complex, respectively (Amelkina et al., 2019). From these, the first population may be homologous with KP neurons of the rodent anteroventral periventricular nucleus which are activated during positive feedback (Herbison, 2008). On the other hand, the role of KP neurons in the cat amygdala is less clear and it was proposed that these KP neurons do not correspond to KP cells described in the medial amygdala in rodents (Amelkina et al., 2019).

Kisspeptin neurons of the anterior periventricular nucleus and the amygdaloid complex remained undetected in our present study. This is most likely explained by the use of OVX animals, in view that the expression of *Kiss1* is positively regulated by estrogens both in the preoptic region (Adachi et al., 2007) and the amygdala (Stephens and Kauffman, 2017). In the context of ovulation, it is important to note that the cat is a reflex ovulator species in which the preovulatory GnRH/LH surge is induced by the receipt of genital somatosensory stimuli during mating. In such species, activation of brainstem noradrenergic neurons is thought to act in the mediobasal hypothalamus to promote GnRH release from nerve terminals in the ME (Bakker and Baum, 2000). While induced ovulating species generally do not show steroid-induced preovulatory LH surges, the cat seems to be an exception in this respect because group-housed females are capable of ovulating spontaneously (Gudermuth et al., 1997). It remains to be clarified how the hypothalamic KP and GnRH neuronal systems of the cat are integrated into the neuronal networks regulating spontaneous *vs.* reflex ovulation.

### Connections Between Kisspeptin Cells and GnRH Neurons in Dogs and Cats

Kisspeptin input to GnRH neurons is critical for reproduction in mice (Kirilov et al., 2013) and humans (de Roux et al., 2003; Seminara et al., 2003), and unlikely to be less important in other mammals like dogs and cats. KP fibers in different species innervate the somatodendritic compartment of GnRH neurons (Kinoshita et al., 2005; Clarkson and Herbison, 2006; Ramaswamy et al., 2008; Smith et al., 2008; Hrabovszky et al., 2010). In mice, these inputs tend to arise from the preoptic region because most of them are devoid of the arcuate nucleus marker NKB (Kallo et al., 2012). The excitatory responses of GnRH neurons to KP (Han et al., 2005; Dumalska et al., 2008; Pielecka-Fortuna et al., 2008) are likely mediated by somatodendritic Kiss1r during positive estrogen feedback. Humans whose GnRH neurons are rather scattered within the hypothalamus (Dudas et al., 2000) differ from mice in that at least 10–30% of their KP afferents contain the INF marker NKB (Hrabovszky et al., 2011; Molnar et al., 2012). In our present study on OVX dogs and cats, the cell bodies and dendrites of GnRH neurons received abundant innervation from KP axons. In dogs, successful detection of NKB signal in over 90% of KP-IR afferents also allowed us to conclude that a high number of these KP inputs originate in the INF. In contrast, lack of sufficient axonal signal for NKB and Dyn within KP axons of the cat did not allow us to firmly establish the importance of KP inputs arising from the INF in this species.

It is known that KP-IR neurons of the mediobasal hypothalamus also establish axo-axonal contacts with hypophysiotropic GnRH fiber varicosities in the ME of various species (Ramaswamy et al., 2008; Krajewski et al., 2010; Matsuyama et al., 2011; Borsay et al., 2014). Kisspeptin likely binds to Kiss1r on these GnRH fibers when inducing GnRH release from mediobasal hypothalamic explants (which contain only a few GnRH cell bodies in rodents) in an action potential-independent manner (d’Anglemont de Tassigny et al., 2008). Systemic KP injection is also thought to stimulate LH secretion via acting on these processes (Matsui et al., 2004; Dhillo et al., 2005; Navarro et al., 2005; Shahab et al., 2005). Finally, axo-axonal contacts may also play a paramount role in the communication between the putative pulse generator KNDy neurons and the GnRH neurosecretory system. Temporal correlation between the pulsatile KP output and GnRH secretory pulses in the ME of monkeys provide strong support for this notion (Keen et al., 2008). Results of our present study established that, similarly to other species, dogs and cats also contain axo-axonal appositions between GnRH-IR and KP-IR fibers in the ME. In addition to these *bona fide* axo-axonal appositions, it remains possible that KP axons also innervate more proximal segments of GnRH fibers before they spray into terminal axon branches. In mice, GnRH fibers bear mixed dendritic and axonal properties (called “dendrons”) and at the border of the ME they receive a particularly high density of synaptic inputs. This connectivity may represent an important anatomical pathway in the regulation of episodic GnRH/LH secretion (Moore et al., 2018). The existence of such GnRH dendrons and their synaptic regulation in other species, including cats and dogs, will require clarification.

### Species Differences in the Neurochemistry of Kisspeptin Cells

The most important message of our present study is the unique neurochemistry of canine and feline mediobasal hypothalamic KP neurons, with both similarities to and differences from the KP system of other species.

Similarities to rodents (Navarro et al., 2009) and the sheep (Goodman et al., 2007) include the co-expression of the three “KNDy” neuropeptides in the same cells. This evolutionarily conserved phenomenon suggests that all three neuropeptides play obligate roles in the generation of GnRH/LH pulses. However, it is worth to note that KP neurons in the INF of *postmortem* human brains only show partial overlap between the KNDy neuropeptides which also varies considerably with sex and age. For example, only ∼36% of NKB neurons express KP immunoreactivity in young human males (Hrabovszky et al., 2012; Molnar et al., 2012). In addition, Dyn immunoreactivity can only be detected very rarely in human KP and NKB neurons and their processes (Hrabovszky et al., 2012, 2019; Skrapits et al., 2015a), in contrast with much higher degrees of co-expression in sheep (Goodman et al., 2007) and mice (Navarro et al., 2009). An interesting similarity between dogs and cats and the human is the relatively high abundance of NKB neurons without KP signal, whereas most KP neurons in all three species contain NKB signal. Our immunofluorescence results on OVX dogs and cats indicate that about ∼25% of labeled neurons in the canine and feline INF are triple-labeled (“KNDy”) neurons, supporting the notion that the overlap of the three KNDy peptides might be functionally important, although far from being complete. It will be important to more thoroughly investigate the relationship between the reproductive status and the co-synthesis/co-secretion of each KNDy peptide in different species.

In addition to identifying the above species similarities, we also observed clear differences between OVX cats and dogs and the more extensively studied rodent and primate species. As opposed to mice (True et al., 2013) and similarly to humans (Hrabovszky et al., 2013; Skrapits et al., 2014), OVX cats showed a considerable co-expression of CART and SP in KP and NKB neurons. Interestingly, the two carnivores differed from each other in this context; canine KP and NKB neurons did not co-contain CART signal, whereas feline KP and NKB neurons did. Similarly, we did not observe SP signal in KP and NKB neurons in OVX dogs. Despite this negative finding, SP co-synthesis likely plays some role in the physiology of these neurons in this species because a gonadally intact male dog included in our study showed SP signal in many KP and NKB neurons. This finding raises the possibility that sex and/or the gonadal steroid status exert critical effects on the SP co-synthesis of KP neurons. Finally, galanin, which has only been observed in murine KP neurons (Porteous et al., 2011; Kallo et al., 2012), was absent from KP cells of both dogs and cats which is similar to our earlier observation on human KP neurons (Skrapits et al., 2015a). Neurochemical differences and common features of different species suggest that some neuropeptides play facultative roles in the generation or fine-tuning of GnRH/LH pulses, whereas the role of others is evolutionarily more conserved. One should keep in mind that colocalization results from different species were obtained using different antibodies and different immunohistochemical techniques. Therefore, comparison of the exact colocalization percentages has innate pitfalls.

## Conclusion

In this study we characterize the distribution of GnRH and KP neurons in the hypothalamus of OVX dogs and cats and identify conspicuous anatomical similarities to and differences from laboratory rodents and primates. We show that the majority of GnRH neurons receive input from KP neurons. The vast majority of these inputs, at least in OVX dogs, originate from KP neurons of the INF. Finally, we determine the unique neurochemistry of KP and NKB neurons in the INF of OVX dogs and cats. Results of immunofluorescent colocalization studies indicate that the three KNDy peptides show partial co-expression, with ∼25% of neurons expressing all three substances. Galanin, observed earlier in murine KNDy neurons only, is absent from canine and feline KNDy neurons. SP, localized previously to human KP neurons but not to rodent KP cells, is present in subsets of KP and NKB neurons in OVX cats but not in OVX dogs, although KP and NKB neurons of a gonadally intact male dog also synthesized SP. Finally, CART, identified earlier as a cotransmitter of KP cells both in human and in non-human primates, occurs in KP and NKB neurons of OVX cats but not OVX dogs.

Anatomical and neurochemical similarities to and differences from homologous and more extensively investigated rodent, domestic and primate KP cells will contribute to our understanding of obligate and facultative players in the molecular mechanisms underlying episodic GnRH/LH secretion in mammals.

## Data Availability Statement

The raw data supporting the conclusions of this article will be made available by the authors, without undue reservation.

## Ethics Statement

The animal study was reviewed and approved by Animal Welfare Committee of the Institute of Experimental Medicine, Institute of Experimental Medicine, Budapest, Hungary. Written informed consent for participation was not obtained from the owners because all pets were euthanized by an authorized veterinarian because of incurable and severe health issues. No animal sacrifice was needed to carry out these studies.

## Author Contributions

KS supervised the work. KS, ÉR, and EH wrote the manuscript with support from all listed co-authors. Tissue samples were provided by FB, OS, and AH. PC provided important reagents. All authors were involved in conceiving the experiments.

## Conflict of Interest

The authors declare that the research was conducted in the absence of any commercial or financial relationships that could be construed as a potential conflict of interest.
